# Pull-to-center is not just for newsvendors

**DOI:** 10.1371/journal.pone.0264183

**Published:** 2022-02-22

**Authors:** Zuzana Brokesova, Cary Deck, Jana Peliova

**Affiliations:** 1 Department of Insurance, Faculty of National Economy, University of Economics in Bratislava, Bratislava, Slovak Republic; 2 Department of Economics, Finance, & Legal Studies, Culverhouse College of Business, University of Alabama, Tuscaloosa, Alabama, United States of America; 3 Department of Finance, Faculty of National Economy, University of Economics in Bratislava, Bratislava, Slovak Republic; Wageningen University, NETHERLANDS

## Abstract

The pull-to-center effect is a systematically observed suboptimal behavior in newsvendor experiments. Various explanations have been forward for this phenomenon, some of which are based on structural properties of the task while others are based upon the inventory context of the problem. To help distinguish between these two types of explanations, we compare behavior in a newsvendor game to behavior in a new, mathematically isomorphic, price gouging game. Our laboratory experiments replicate the standard results for newsvendors and yield the equivalent pattern in the price gouging game. This suggests previously observed newsvendor behavior is driven by structural aspects of the task consistent with models like prospect theory and impulse balance rather than context specific explanations pertaining to inventory management.

## Introduction

The newsvendor problem is perhaps the simplest inventory decision setting–a newsvendor orders inventory at a set cost per unit to be resold at a pre-specified price before the quantity demanded is determined. The theoretically optimal solution for an expected profit maximizing newsvendor has been known since [1]. However, starting with [2], controlled laboratory experiments have consistently demonstrated behavior that systematically differs from the optimal inventory order. In their chapter on inventory behavior in *The Handbook of Behavioral Operations*, [3] (p. 427) note “*the pull-to-center effect is the dominant observation in previous experimental studies*.” Pull-to-center is the term that has been given to the observation that people order too few units when the per unit inventory cost is relatively low and too many units when the cost is relatively high. Further, the magnitude of the pull-to-center effect is usually found to be asymmetric, pulling stronger when inventory levels are predicted to be higher [4].

In their surveys of the literature, [5] as well as [6] highlight that that the pull-to-center phenomenon is robust to different cost parameters, demand distributions, learning schemes, procurement experiences, types of information provided to participants, and even subject culture. More recently, [7] confirm the pull-to center effect in newsvendor decisions under demand ambiguity and [8] suggest the pull-to-center effect is an aggregate data phenomenon, but point out that individual decision makers are highly heterogeneous. Given this compelling evidence of a pull-to-center effect in the newsvendor game, scholars have investigated a wide array of potential drivers of the pull-to-center effect. [9] offer a systematic review of literature for explanations for the pull-to-center effect in operations management. The pattern is not consistent with explanations such as risk aversion or loss aversion, nor is it consistent with some inventory context specific factors such as stock out aversion or waste aversion [2, 10–12], although [13] do find a significant correlation between individual risk preferences and order quantities. However, evidence has been found in support of other explanations. For example, [14] find evidence in favor of an inventory context explanation as they report that subjects behave as if they have a preference to reduce ex-post inventory error. In contrast, [15] argue that pull-to-center is driven by impulse balance while [16] argue it is explained by prospect theory, both of which are based on the underlying structure of the task in terms of payoffs and probabilities and neither of which is dependent upon the inventory setting context.

The goal of this paper is to determine if the typically observed pattern of behavior arises in non-inventory management settings. While there have been some previous experiments varying how the newsvendor task is framed, these studies have generally maintained the inventory setting. For example, [17] present an inventory shortfall as either not being able to meet realized demand, resulting in an opportunity cost of missed sales or a shortage cost for having to rush order additional inventory after the demand in known. [18] vary the saliency of the revenue and loss aspects of the task. [19] describe the task as a revenue management problem where the subject decides how to allocate inventory across two markets—a low value and a high value market—with the catch being the low value market moves first and the size of the high value market is unknown. These studies document that the pull-to-center effect is robust within the inventory context but cannot identify if a similar pattern emerges in non-inventory management settings.

There are at least two studies that consider what is structurally a newsvendor problem in an abstract context free setting, but they report contradictory evidence. Specifically, [14] report that behavior differs between the inventory management framed newsvendor game and the neutrally framed version suggesting context dependent behavior. However, there are several features of [14] that warrant noting: demand realizations and inventory quantities were limited to only seven levels; subjects were given a complete payoff table for all choices; in the inventory context subjects were not given the price and unit cost parameters. Further, subjects only experienced 30 periods in a treatment and the sample size was small. More recently, using a large sample [20] found no significant behavioral differences between inventory management framed and neutrally framed versions of the task. However, the subjects in [20] were all taking a course in operations management and received extra credit for participation, either of which may have impacted the findings.

To further explore the robustness of the pull-to-center effect in an effort to determine if it is inherent to the underlying structure of the task as posited by explanations such as prospect theory or impulse balance or an artifact of the inventory management context, we introduce a new game which we refer to as the price gouging game. The price gouging game is functionally distinct from the newsvendor game, but under certain restrictions, the two games can be made isomorphic. With our between subjects experimental design, we find that behavior in the two games is quite similar including the asymmetry in the strength of the pull-to-center, indicating that the effect is not due to the specific context but rather driven by the underlying aspects of the decision problem.

## Method

### The newsvendor game

The newsvendor problem is to decide how much inventory to hold prior to the quantity demanded being realized when the price is fixed. Because the newsvendor has to pay for each unit of inventory regardless of whether or not it is sold, the newsvendor has to balance the excess cost of acquiring too much inventory and the missed opportunity to make profitable sales from not holding enough inventory. Formally, let *p* be the price, *c*>0 be the per unit inventory cost (with *p*>*c*, and *f*() be the distribution determining the quantity demanded. The newsvendor’s objective is to select the inventory level, q*, that maximizes her expected profit given by Eq (1).


$$(1-F(q))(p-c)q+\int_{o}^{q}\left(px-cq)f(x\right)\;dx$$ (1)

The solution, due to [1], is such that $F(q^{*})=\frac{p-c}{p}$.

### The price gouging game

Price gouging is defined by [21] (p. 347) as occurring when *“in the wake of an emergency*, *sellers of certain necessary goods sharply raise their prices beyond the level needed to cover increased costs*.*”* For example, after Hurricane Irma in Florida and Harvey in Texas, both packs of bottled water and hotel rooms became three or four times more expensive. While a significant increase in price is the expected market outcome when demand spikes and sellers are unable to adjust inventory, as is the case in the aftermath of a hurricane, the practice of price gouging is often viewed as being morally and ethically questionable. As a result, many locations have enacted laws to prohibit sellers from charging higher prices, especially in states of emergency. Sellers who are found to have engaged in price gouging often face stiff fines. Thus, sellers in such settings face the following optimization problem. The seller can raise the price of her inventory to earn a larger profit. However, as she raises her price, she increases the chance of being deemed a price gouger and having to pay penalties. Hence, the seller’s profit problem is to charge the highest price possible without being identified as price gouging.

Before continuing, we want to emphasize that our focus is not on price gouging per se, but rather on leveraging such a setting to better understand behavior in the newsvendor game. Although price gouging as it relates to supply chain responses to catastrophic events is an important issue and worthy of detailed study, we omit many features such as seller reputation and consumer backlash that may be relevant in actual price gouging settings. Of course, models of the newsvendor typically ignore the negative reputational impact on a vendor who regularly runs out of inventory.

To model price gouging, we assume that a seller has a fixed inventory of *θ*>0 units available and sets her price, *π*. The seller knows all of the units can be sold regardless of the price charged due to the spike in demand. Thus, the seller’s revenue is *πθ*. What the seller does not know is the threshold price, *τ*>0, which will trigger a penalty for price gauging. In some jurisdictions what price changes constitutes price gouging is defined in terms of a percentage mark-up over previous prices, but in other jurisdictions what constitutes price gouging is vague and depends on interpretation of exorbitant or excessive price increases. However, even in the former case the portion of a price increase that can be justified by a cost increase may still be a matter of prosecutorial discretion. But it is assumed that the seller does know that the threshold is distributed according to *ϕ*(), with cumulative distribution function Φ(). For the sake of comparison with the newsvendor, the support of *ϕ*() starts at 0. Without loss of generality, the lower bound can be viewed as the normal (pre-emergency) price and the upper bound viewed as the (post-emergency) mark-up customers would be willing to pay. There are many ways that a penalty could be implemented and some may be more or less reflective of government behavior in different locations. For example, a seller that is found to be engaging in price gouging could be forced to pay a fixed fine. However, for our purpose of better understanding newsvendor behavior, we assume the penalty is proportional to the difference between the price set by the seller and the threshold price. Formally, the penalty is *γ*(*π*−*τ*) if the price exceeds the threshold (i.e. *π*>*τ*) where *γ* is the fixed penalty per dollar charged in excess of the threshold. Therefore, if *π*≤*τ* the seller’s profit is *πθ* and if *π*>*τ* the seller’s profit is *θ*−*γ*(*π*−*τ*). We assume that *γ*>*θ* so that the penalty from price gouging exceeds the revenue from price gouging as otherwise the seller would always set the maximum price and simply pay the (random) fine. This penalty structure puts downward pressure on price as a seller wants to avoid the penalty similar to the downward pressure on inventory that overage costs exert on newsvendors. Of course, for each dollar amount the price falls below the threshold the seller has the opportunity cost of foregone profit in the same way that a newsvendor who does not order enough units to meet demand experiences an underage (opportunity) cost. The price gouger’s objective is thus to select *π*, so as to maximize expected profit, which is given by Eq (2).


$$(1-\Phi (\pi ))\pi \theta +\int_{0}^{\pi }\left(\pi \theta -\gamma (\pi -x))\phi (x\right)\;dx$$ (2)

This objective function in (2) is similar to that of the newsvendor in (1). A low price limits the upside profit potential, but also limits downside losses, similar to a low inventory selection by the newsvendor. Likewise, charging a high price creates the potential for a large payoff, but also exposes the seller to a large potential loss just as selecting a high inventory level does for the newsvendor. The solution to the price gouger’s problem in (2) is to set a price of π* such that $\Phi (\pi^{*})=\frac{\theta }{\gamma }$.

**Fig 1:**
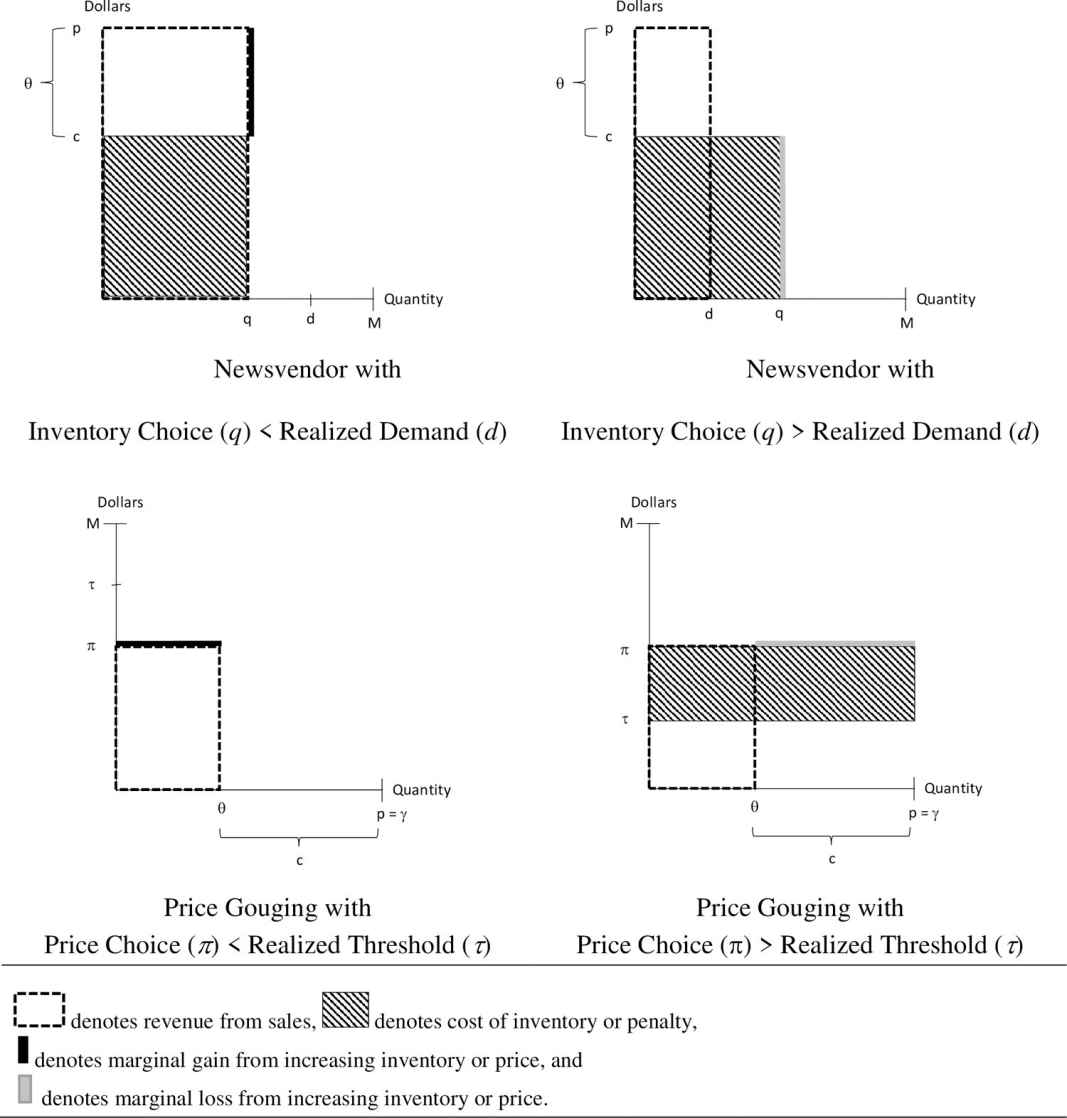
Comparison of newsvendor and price gouging problems.

Consider a newsvendor facing a demand, *d*~*U*[0,*M*]. The upper portion of Fig 1 shows the revenue and inventory costs to the newsvendor who has a set price of p. The upper left panel is for a realized demand in excess of the ordered inventory level and the upper right panel is for a realized demand less than the inventory level. Careful choice of *γ*, *θ* and *ϕ*() can make the price gouging game isomorphic to the newsvendor game in the sense of there being a one-to-one correspondence between choices and payoffs. The lower portion of Fig 1 shows the same situations in a price gouging game with *γ* = *p*, *θ* = *p*−*c* and *τ*~*U*[0,*M*]. The lower left panel shows a set price below the realized threshold and the lower right panel shows the case when the price exceeds the threshold. With these constraints, the two tasks become isomorphic in terms of the equivalent action yielding the same payoff for the corresponding draws from the relevant random variable. That is, for each choice in the newsvendor game, a price gouger has a corresponding action that generates the same payoff distribution. Hence, pairs of corresponding actions yield the same expected payoff so that the risk neutral optimal choice is the same. Further, even agents who are risk averse as in [22] will make the same choices across games. Similarly, agents whose preferences are described by prospect theory, or any other model that depends only on payoffs and probabilities, would select the same action in both games (e.g. [23]). An alternative way to see the equivalence is to compare the payoff impact from a change in the choice variable. For the newsvendor, a unit increase in the choice variable (*q*) generates an additional revenue of *p* and cost of *c* for a marginal profit of *p*−*c*, so long as *q* is less than the randomly generated *d*, the thick black line in the top left panel of Fig 1. For the price gouger, a unit increase in the choice variable (*π*) generates an additional profit of *θ* so long as *π* is less than the randomly generated *τ*, the thick black line in the bottom left panel of Fig 1.

When the newsvendor’s choice of *q* exceeds the randomly generated *d*, then a unit increase in the choice variable results in a loss of *c*, the thick gray line in the top right panel of Fig 1. When the price gouger’s choice of *π* exceeds the randomly generated *τ*, then a unit increase in the choice variable results in a loss of *γ*−*θ*, the thick gray line in the lower right panel of Fig 1.

Fig 1 also highlights the sense in which price gouging and newsvending are distinct. In the newsvendor game, the seller’s cost is determined by her decision. Thus, the cost of inventory (the hashed boxes) in the upper portion of Fig 1 are identical. What is uncertain is the revenue. By contrast, in the price gouging game the seller’s revenue is determined by her decision and it is cost that is uncertain. Thus, the revenue from sales (the boxes with dashed borders) in the lower portion of Fig 1 are identical.

### Design

We conducted a 2 × 2 between subjects experimental design. The first dimension is the structure of the game: newsvending or price gouging. The second dimension is the cost (*c* in the newsvending game) or the inventory level (*θ* in the price gouging game): either low or high.

For the newsvending game, *p* = 100 and *d*~*U*[0,100]. In the low cost treatment, *c* = 25 and thus $q_{Low}^{*}=75$. In the high cost treatment, *c* = 75 and thus $q_{High}^{*}=25$. To maintain an isomorphism between game structures, *γ* = 100 and *τ*~*U*[0,100] in the price gouging game. For the low inventory treatment, *θ* = 25 and thus $\pi_{Low}^{*}=25$. For the high inventory treatment, *θ* = 75 and thus $\pi_{High}^{*}=75$. Formally, our experiment tests if subjects make the optimal choice in each of the four treatments versus the alternative that observed choices exhibit the pull-to-center effect. Further, the experiment also tests if theoretically isomorphic games yield observationally equivalent behavior. If models such as impulse balance or prospect theory explain pull-to-center in the newsvendor game then behavior should be the same in the low cost (newsvendor) and the high inventory (price gouging) treatments and behavior should be the same in the high cost (newsvendor) and the low inventory (price gouging) treatments. However, if pull-to-center is being driven by the operations context then we would not expect to see similar differences in each pair of treatments.

The expected profits from optimal decision making differ between the low cost/inventory and high cost/inventory treatments. Further, it is possible that participants could lose money depending on their choice and the realization of demand/threshold. To account for these potential incentive issues, we provide treatment specific endowments that keep the optimal profit constant across treatments. For the low cost/high inventory treatments, the endowment was set at 300. For the high cost/low inventory treatments, the endowment was set at 2,000.

Subjects first read computerized instructions and then completed a series of comprehension questions. Copies of the instructions, which closely mimic those of [20], are available in the supporting material (see S1 File). Aside from the necessary changes regarding choice variables and how profits are determined, the two sets of instructions are parallel to avoid introducing a confound into the experiment. Subjects may have preconceived ideas about price gouging, which is often portrayed in the news as unethical or immoral despite it often being welfare improving. Thus, the instructions for the price gouging treatments intentionally do not mention the term “price gouging” but rather refer to setting a price. While such moral concerns may be of great importance for price gouging in practice, the goal of this paper is not to study price gouging per se, but rather to provide a different context for examining the same decision task as that faced by a newsvendor. This is critical given that our main focus is to determine if the pull-to-center effect is robust to the decision context.

In the experiment, subjects completed 100 decision periods in the assigned treatment. The realized demand or the price gouging threshold was randomly drawn for each subject each period. After each period, a subject received feedback in the form of the realized demand or threshold as well as the subject’s profit. A table that was always visible on the subject’s screen contained the feedback from all completed periods. All payoff amounts are denoted in Experimental Currency Units (ECUs). Subjects were paid their cumulative earnings, which were converted into $US at the rate 12,500 ECU = 1 $US.

After completing the decision periods and before receiving their payoffs, subjects also completed a short survey consisting of a question regarding the subject’s gender, the cognitive reflection task (CRT), and a non-incentivized risk assessment. The cognitive reflection task consisted of six question drawn from [24] and [25]. Each of these questions has an intuitive, but incorrect, response and together the questions are designed to measure how deeply a subject is willing to think before responding. The risk assessment is derived from [26], but uses US dollar amounts rather than Euros. It states “*Suppose that you earned $100*,*000 in lottery winnings*. *How much of the $100*,*000 would you be willing to invest in an asset to either HALVE or DOUBLE in two years’ time with equal probability*?” These survey questions allow us to control for individual characteristics that prior research has suggested is associated with behavior in the newsvendor game. For example, [27] observe gender differences in newsvendor behavior. With respect to cognitive reflection, [28] and [29] report that subjects who score higher on the CRT have lower forecast errors and are less likely to engage in demand chasing than those who score lower on the CRT. Finally, with respect to risk attitudes, [30] and [31] show that a risk-averse newsvendor should order less inventory that the expected profit-maximizing choice.

### Participants

The study was conducted in TIDE Lab at the University of Alabama. The study was approved by the Institutional Review Board at the University of Alabama (protocol 18-02-921). Written consent was obtained from each participant.

A total of 105 subjects completed the study. All of the subjects were undergraduate students at The University of Alabama and had previously volunteered to be in the lab’s standing subject pool. Approximately two-thirds of the subject pool consisted of students in the business school with the remainder being mostly arts and science or engineering students. While some of the subjects had participated in unrelated studies, none had participated in a previous newsvendor experiment. Fifty-two percent of the subjects were male. The average number of CRT questions answered correctly was 2.43 and the average amount invested in the risk assessment was 38%. Table 1 compares these characteristics by treatment. None of these characteristics differed significantly between any pair of treatments (all 18 p-values greater than 0.05). The average earnings for the 30 minute sessions were $23.40 including a $5.00 participation payment. Initially participants were recruited for a 60-minute session, but on average people finished in 30 minutes. Because the instructions and survey typically took less than 5 minutes, this means that subjects were taking about 15 seconds to review the feedback for one period and make their choice for the next period. While this may seem like a short time, we encourage the reader to contemplate it for 15 seconds.

**Table 1:** Participant characteristics by treatment.

	Newsvending		Price gouging	
	Low cost	High cost	High inventory	Low inventory^a^
Participants	26	26	26	27
Percent male	46%	62%	46%	56%
Average cognitive reflection score	1.92	2.65	2.62	2.52
Average amount invested in risky asset	37%	35%	40%	38%

a. One subject in the low inventory price gouging treatment entered a price of zero in every period. In the data analysis in the next section, the person who entered a price of zero every period has been excluded although the results remain qualitatively similar if that observation is included. An additional observation was collected for this treatment to balance the design.

## Results

For both the low inventory cost newsvending game and the high inventory level price gouging game, the optimal quantity and price choice is 75, respectively. For the high inventory cost newsvending game and the low inventory level price gouging game the optimal price and quantity choice is 25, respectively. Table 2 gives the descriptive statistics for each treatment. For none of the four treatments is the observed mean statistically equal to the optimal value and in each case the observed mean exhibits a pull-to-center effect. The data necessary to conduct these tests and all other data related to this study can be found in the supporting materials (see S1 Raw data).

**Table 2:** Descriptive statistics of inventory and price choices.

	Optimal	Average inventory or average price	Within subject standard deviation	Number/Percent demand chasing or price chasing
Newsvending				
Low cost	75	**53.92**	9.87	13 / 50%
		(19.79)	(5.91)	
		[<0.01]		
High cost	25	**42.67**	10.37	17 / 65%
		(17.03)	(6.69)	
		[<0.01]		
Price gouging				
High inventory	75	**51.56**	13.45	12 / 46%
		(16.60)	(6.47)	
		[<0.01]		
Low inventory	25	**41.30**	9.74	7 / 27%
		(16.59)	(7.00)	
		[<0.01]		

Note: Ex-post power analysis for the average inventory (price) being different from the optimal level indicates that the data provide power > 0.99 in all four treatments when using the standard value of α = 0.05. Unit of observation is a single participant and thus each average and standard deviation is based on 26 observations. Standard deviation of measurement given in parentheses. p-values for testing two-sided t-test of difference between mean and optimal behavior are in brackets. Bold coefficients denote significant difference from 0 at the 0.05 level.

*Finding 1. Both newsvendor inventory choices and price gouging price choices differ from the optimal behavior and exhibit a pull-to-center effect*.

While not our primary focus, to explore the determinants of subject choices in more detail, Table 3 reports regression analysis where the dependent variable is the average choice by a subject and individual characteristics as measured by the post-study survey are included to control for between subject variation. Formally, the variable *Male* takes the value one if the subjects was male and is zero otherwise; *CRTSc* is the number of correct responses given by the subject on the CRT; and *Risk* is the percentage of money the subject was willing to invest in a hypothetical risky asset. Separate regression analysis is conducted for each of the four treatments. To allow for the possibility that individual characteristics may only impact behavior initially or alternatively only after a decision maker has some familiarity with the task, the analysis is conducted for all 100 periods (columns 1, 3, 5 and 7 in Table 3) and for only the last quarter of the decisions (columns 2, 4, 6 and 8 in Table 3). In Table 3 the dependent variable is the average response by an individual for the stated number of periods. Because each subject account for a single observation, we rely upon ordinary least squares regression. The results indicate that gender, cognitive reflection, and risk attitude do not play a major role in explaining variation in behavior; however, these results should be treated as exploratory as we did not have *a priori* hypotheses regarding these characteristics.

**Table 3:** Regression analysis of individual characteristics on average choice.

	Newsvending				Price gouging			
	Low cost		High cost		High inventory		Low inventory	
Periods	1–100	75–100	1–100	75–100	1–100	75–100	1–100	75–100
*Constant*	**45.13**	**44.42**	**34.26**	**31.93**	**40.81**	**38.44**	**43.16**	**44.36**
	(5.07)	(7.37)	(4.51)	(4.88)	(5.73)	(6.68)	(4.85)	(5.41)
	[<0.01]	[<0.01]	[<0.01]	[<0.01]	[<0.01]	[<0.01]	[<0.01]	[<0.01]
*Male*	-4.58	-4.38	-0.21	1.56	-7.40	-8.95	2.82	3.60
	(4.13)	(6.00)	(4.23)	(4.57)	(4.97)	(5.80)	(4.24)	(4.73)
	[0.28]	[0.47]	[0.96]	[0.73]	[0.15]	[0.14]	[0.51]	[0.45]
*CRTSc*	1.89	2.45	2.26	**2.84**	3.20	**4.61**	-0.14	-0.71
	(1.59)	(2.31)	(1.17)	(1.26)	(1.55)	(1.81)	(1.21)	(1.34)
	[0.25]	[0.30]	[0.07]	[0.04]	[0.51]	[0.02]	[0.91]	[0.60]
*Risk*	**0.20**	**0.26**	0.07	0.05	0.18	**0.26**	-0.07	-0.09
	(0.09)	(0.12)	(0.10)	(0.11)	(0.10)	(0.11)	(0.08)	(0.09)
	[0.03]	[0.05]	[0.47]	[0.73]	[0.08]	[0.03]	[0.41]	[0.36]
Observations	26	26	26	26	26	26	26	26

Note: Standard errors are in parentheses. p-values are in brackets. Bold coefficients denote significant difference from 0 at the 0.05 level.

**Fig 2:**
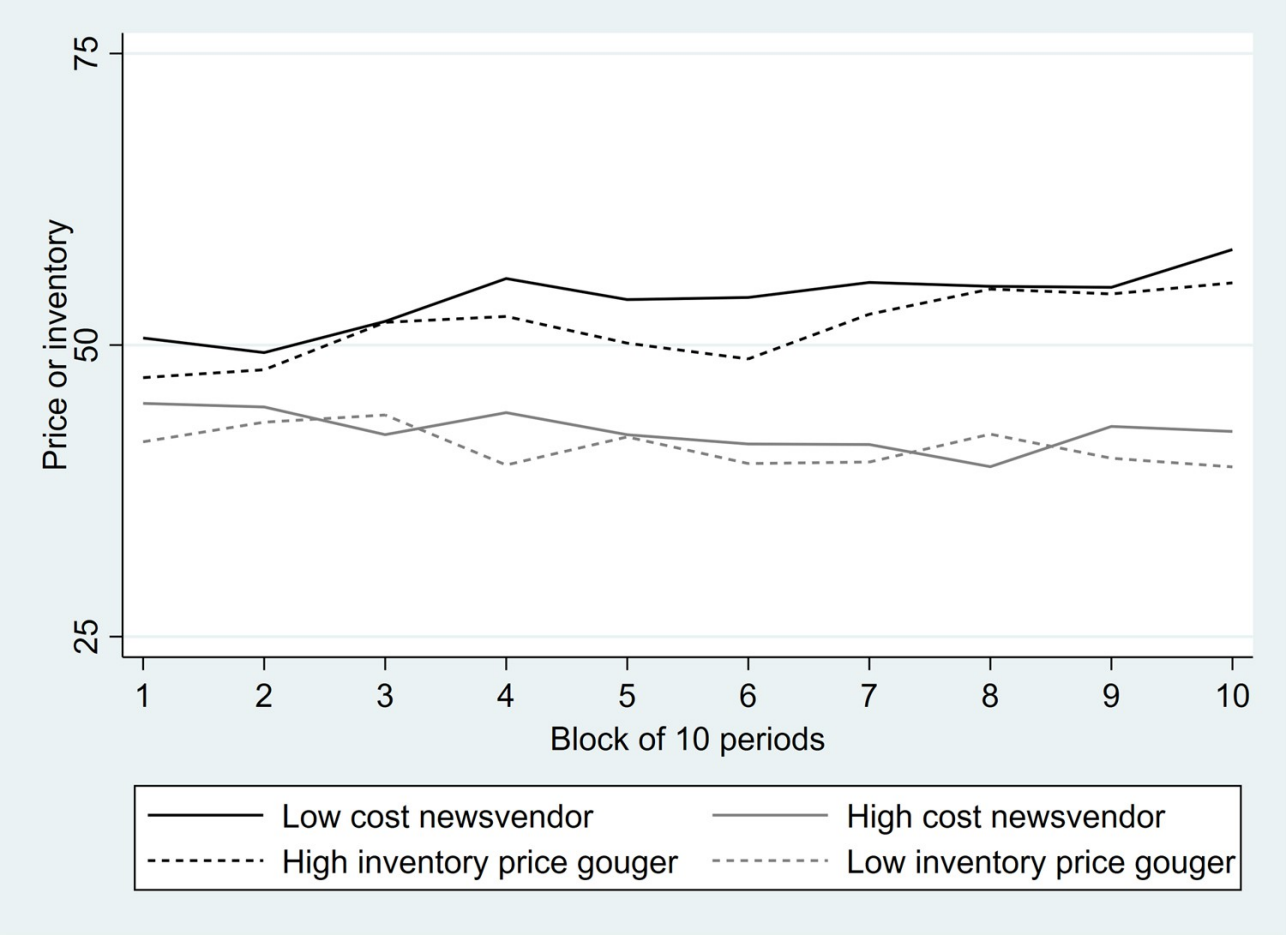
Inventory and pricing decisions over the experiment.

Fig 2 plots average behavior over the course of the experiment by treatment in blocks of ten periods with the pull-to-center effect readily apparent. Of course, there is considerable heterogeneity across subjects. In the supporting material, we provide plots grouped by treatment of individual level behavior across all 100 periods of the experiment (see S1 Text and S1–S4 Figs). Only four subjects make average decisions that are not statistically different from the optimal level while the other 96% do not make optimal choices on average. Of the 100 subjects who do not make optimal choices, 66 exhibited the pull-to-center effect as their average response fell between 50 and the profit maximizing choice. Of the others, the average choice was more extreme than the optimal for 3 subjects and the average choice was on the opposite side of 50 from the optimal choice for the other 31 subjects. Thus, among our subjects there is considerable evidence of the pull-to-center effect even at the individual level.

Fig 2 reveals two additional important patterns. First, there is little difference in average choices between isomorphic versions of the newsvendor and price gouging games, although subjects are slower to raise their prices in the high inventory price gouging game than they are to increase inventory in the low cost newsvendor game. Second, there is an asymmetry in the strength of the pull-to-center effect with those subjects predicted to select a high inventory level (or price) being closer to the mean demand (or threshold). These two patterns are examined in more detail below.

Table 4 reports the results of panel regression analysis of period level subject responses with random effects for each subject that compares theoretically isomorphic newsvending and price gouging games. The dependent variable is the choice of a subject in a period. The first and second columns compare behavior in the low inventory cost newsvendor game and the high inventory price gouging game. The third and fourth columns compare behavior in the high inventory cost newsvendor game and the low inventory price gouging game. For both pairs of isomorphic games, the coefficient on *PriceGouging*, which is an indicator function for observations from the price gouging game, is not significantly different from zero. This result is shown in the first and third columns of Table 4. The lack of a difference between the two contexts is robust to inclusion of a time trend in the data as shown in the second and fourth columns of Table 4. The positive significant coefficient for *Period* in column 2 of Table 4 reveals that decisions improve with experience in the two relevant treatments (i.e. newsvendor inventory is increasing over time when inventory cost is low and price gouger prices are increasing over time when the fixed inventory level is high). The negative significant coefficient for *Period* in column 4 of Table 4 indicates decisions are also improving with experience in the other two treatments (i.e. newsvendor inventory is decreasing over time when inventory cost is high and price gouger prices are decreasing over time when the fixed inventory level is low). This improvement is consistent with results reported by [32]. The lack of significance for *Period × PriceGouging* in both column 2 and column 4 of Table 4 indicate that experience does not have a differential effect between contexts in either pair of isomorphic games. The results presented in Table 4 provide the basis for our main finding.

**Table 4:** Analysis of difference between newsvending and price gouging choices.

	Low inventory cost & High inventory level		High inventory cost & Low inventory level	
*Constant*	**53.92**	**50.18**	**42.67**	**44.31**
	(2.31)	(2.401	(1.97)	(2.07)
	[<0.01]	[<0.01]	[<0.01]	[<0.01]
*PriceGouging*	-2.36	-2.86	-1.37	-1.57
	(3.27)	(3.40)	(2.79)	(2.93)
	[0.47]	[0.40]	[0.62]	[0.59]
*Period*	----	**0.074**	----	**-0.03**
	----	(0.01)	----	(0.01)
	----	[<0.01]	----	[0.01]
*Period × PriceGouging*	----	0.01	----	0.00
	----	(0.02)	----	(0.02)
	----	[0.59]	----	[0.83]
Observations	5,200	5,200	5,200	5,200

Note: Standard errors are in parentheses. p-values are in brackets. Bold coefficients denote significant difference from 0 at the 0.05 level.

*Finding 2: Isomorphic versions of the newsvendor and price gouging games yield similar average behavior*.

We now turn to the question of asymmetry in the pull-to-center effect. We follow [20] and [33] in defining an anchoring score as $\left(q_{itc}-q_{c}^{*})/(50-q_{c}^{*}\right)$ for the newsvendor game where *q*_*itc*_ denotes the inventory quantity choice of subject *i* in period *t* experiencing cost *c*. The analogous anchoring score for the price gouging game is $\left(\pi_{jt\theta }-\pi_{\theta }^{*})/(50-\pi_{\theta }^{*}\right)$ where *π*_*jtθ*_ denotes the price choice of subject *j* in period *t* with inventory level *θ*. The 50 in each measure is the mean of the relevant distribution and a * denotes the expected payoff maximizing choice of the given treatment.

Table 5 reports the results of panel regression with random effects for each subject with dependent variable is the anchoring score for a subject in a period. Models regressing the anchoring measure described above on *HighOptimal*, which is an indicator variable that takes the value one for an observation in which the optimal choice exceeds the mean of the relevant distribution and is zero otherwise (i.e. *HighOptimal* equals 1 for the isomorphic low cost newsvendor and the high inventory price gouger games and is zero for the other pair of isomorphic games). As in Table 4, we present a basic speciation (in columns 1, 3 and 5) as well as a specification that allows for time trends (in columns 2, 4, and 6) to evaluate the robustness of the main variable of interest. In the first two specifications of Table 5, we use data from all four treatments. In these two specifications the results indicate that the pull-to-center effect is greater when the optimal choice exceeds the mean of the relevant distribution (i.e. *HighOptimal* is positive and significant in columns 1 and 2). The second and third pair of specifications reported in Table 5 repeat this same analysis for each context separately. Again, the results are generally consistent with an asymmetric pull-to-center effect albeit more weakly. The results in Table 5 provide the basis for our next finding.

**Table 5:** Analysis of asymmetric pull-to-center effect.

	Combined data		Newsvending		Price gouging	
*Constant*	**0.68**	**0.74**	**0.71**	**0.77**	**0.65**	**0.71**
	(0.06)	(0.06)	(0.08)	(0.08)	(0.09)	(0.10)
	[<0.01]	[<0.01]	[<0.01]	[<0.01]	[<0.01]	[<0.01]
*HighOptimal*	**0.21**	**0.31**	0.14	**0.22**	**0.29**	**0.40**
	(0.09)	(0.09)	(0.11)	(0.12)	(0.13)	(0.14)
	[0.01]	[<0.01]	[0.23]	[0.06]	[0.03]	[<0.01]
*Period*	---	**-0.00**	---	**-0.00**	---	**-0.00**
	---	(0.00)	---	(0.00)	---	(0.00)
	---	[<0.01]	---	[0.01]	---	[0.02]
*Period ×*	---	**-0.00**	---	**-0.00**	---	**-0.00**
*HighOptimal*	---	(0.00)	---	(0.00)	---	(0.00)
	---	[<0.01]	---	[0.03]	---	[<0.02]
Observations	10,400	10,400	5,200	5,200	5,200	5,200

Note: Standard errors are in parentheses. p-values are in brackets. Bold coefficients denote significant difference from 0 at the 0.05 level.

*Finding 3: The pull-to-center effect is stronger when the optimal choice exceeds the mean of the demand or threshold distribution*.

As shown earlier in Table 2 as well as in the supporting material (S1 Text and S1–S4 Figs), there is considerable heterogeneity in behavior within subject. Table 6 reports exploratory regression analysis that relates the standard deviation of a subject’s own choices to the subject’s characteristics from the post experiment survey. The dependent variable is the standard deviation for a subject over the stated periods. Because each subject accounts for a single observation, we rely upon ordinary least squares regression. The Table 6 considers behavior from the entire experiment as well as separately for the last 25 decision periods as did Table 3. While better performance on the cognitive reflection task is associated with reduced within-subject variation in choices, neither gender nor risk attitude correlate with the within subject variation in behavior.

**Table 6:** Analysis of the within subject variation of choices.

	Periods 1–100	Periods 75–100
*Constant*	**21.07**	**21.30**
	(1.68)	(2.11)
	[<0.01]	[<0.01]
*HighOptimal*	-0.86	0.94
	(1.22)	(1.53)
	[0.48]	[0.54]
*PriceGouging*	-1.55	-1.90
	(1.22)	(1.53)
	[0.21]	[0.22]
*CRTSc*	**-1.22**	**-1.77**
	(0.39)	(0.49)
	[<0.01]	[<0.01]
*Male*	-1.55	-2.15
	(1.29)	(1.62)
	[0.23]	[0.19]
*Risk*	0.02	-0.01
	(0.03)	(0.03)
	[0.57]	[0.74]
Observations	104	104

Note: Standard errors are in parentheses. p-values are in brackets. Bold coefficients denote significant difference from 0 at the 0.05 level.

One potential explanation for the within-subject variation is what [32] refers to as demand chasing, where newsvendors in period *t* are responding to the realized demand in period *t-1* even though demand is independent each period. There are multiple statistics used in extant literature to assess demand chasing. However, as noted by [34] (p. 1248) “*a simple measure of the correlation between the previous demand and the current order quantity does not suffer from [inflated Type I errors]*, *and it is also a reasonably powerful test (when there is true demand chasing)*.” Following this approach, we classify a newsvendor as a demand chaser if the correlation between the subject’s inventory choice and the realized demand from the previous period is significant at the 0.05 level. For price gougers, the analogous price chaser is defined as a subject whose price choice is significantly correlated at the 0.05 level with the penalty threshold in the previous period. In the supporting materials (S1 Text and S1–S4 Figs) we identify which individual subjects meet this definition of chasing, but the right column of Table 2 gives the percentage of demand and price chasing subjects by treatment. From the table, it appears that demand chasing is more prevalent than price threshold chasing and indeed a chi-squared test indicates that the frequency of this behavior is not independent of treatment (p-value = 0.050). This serves as the basis for our final finding. This difference may be driven by understocking and overstocking being natural measures of performance, whereas there is no natural notion of overpricing and underpricing. This may make regret more salient in the newsvendor game and thus lead to greater demand chasing.

*Finding 4: Demand chasing in the newsvendor game is more prevalent than the analogous behavior in the price gouging game*.

Given that an increased CRT score is associated with a reduced variance in choice behavior, one might suspect that subjects with higher CRT score are less likely to chase demand or the price threshold. While [35] reports that higher CRT scores are associated with less demand chasing, [20] report no relationship between demand chasing and CRT score, but do find evidence that women are more likely to chase. They also report that neutral framing of the newsvendor game reduces chasing behavior relative to the framed version of the game. Table 7 reports the results of probit regressions that examine how individual characteristics impact the chance an individual in our sample chases demand or alternatively chases the price threshold. The dependent variable is binary and equals 1 if the subject is classified as a demand/price chaser. Otherwise, it is 0. The first column of Table 7 uses data from all four treatments while the analysis in each of the other columns is based on a single treatment. Our results do not find a statistically significant relationship between the CRT score, gender, or risk attitude on chasing behavior. We do note that the negative and significant coefficient on *PriceGouging* in the first column of Table 7 offers further supporting evidence for Finding 4.

**Table 7:** Probit analysis of chasing behavior.

	Combined data	Newsvending		Price gouging	
		Low cost	High cost	High inventory	Low inventory
*Constant*	**0.70**	1.25	0.82	-0.03	0.53
	(0.32)	(0.78)	(0.60)	(0.60)	(0.86)
	[0.03]	[0.11]	[0.17]	[0.97]	[0.54]
*PriceGouging*	**-0.52**	---	---	---	---
	(0.25)	---	---	---	---
	[0.04]	---	---	---	---
*Male*	-0.31	-0.51	0.08	-0.43	-0.52
	(0.27)	(0.56)	(0.58)	(0.53)	(0.59)
	[0.24]	[0.36]	[0.90]	[0.42]	[0.38]
*CRTSc*	-0.04	-0.24	-0.22	0.06	-0.04
	(0.08)	(0.23)	(0.16)	(0.16)	(0.18)
	[0.59]	[0.29]	[0.18]	[0.73]	[0.82]
*Risk*	-0.01	-0.02	0.00	0.00	-0.02
	(0.01)	(0.01)	(0.01)	(0.01)	(0.02)
	[0.24]	[0.26]	[0.76]	[0.95]	[0.15]
Observations	104	26	26	26	26

Note: Ex-post power analysis for the model with combined data indicates that the data provide power > 0.85% for identifying the effect of game type on chasing behavior using the standard level of α = 0.05. Standard errors are in parentheses. p-values are in brackets. Bold coefficients denote significant difference from 0 at the 0.05 level.

## Discussion

We introduce and experimentally test an alternative setting that shares the underlying structure of the newsvendor problem while introducing a non-inventory decision framework. While a newsvendor has to order costly inventory before demand is known, in our price gouging game a seller faces a penalty from charging a price above an unknown threshold. If behavior differs between the two games, it would suggest that the pull-to-center effect commonly observed in newsvendor experiments is being driven by context specific factors as posited by some researchers. However, if structural models such as prospect theory or impulse balance explain pull-to-center as posited by other researchers, then behavior should be equivalent in the two games.

Using controlled laboratory experiments, we compare newsvending and price gouging behavior. For both games, we find clear evidence of a pull-to-center effect. Further, the pull-to-center effect is stronger when the optimal choice is above the mean of the distribution for demand (in the case of newsvending) or for the penalty threshold (in the case of price gouging). The results also reveal that average choices are similar across the theoretically isomorphic games. This is an important finding as it indicates the behavior commonly observed in newsvendor experiments generalizes to a broader class of games and is not driven by the inventory management framing. In particular, our results suggest that observed behavioral patterns have more to do with people understanding how their choices impact the conditional distribution of outcomes consistent with prospect theory or impulse balance rather than concerns about wasted inventory, stock out aversion, or other context specific notions, per se. As a secondary result, we find little evidence that behavior is influenced by personal characteristics such as gender, risk attitude, or cognitive reflection.

By considering the newsvendor as a special case of a larger family of games, we are able to gain more insight into what is and what is not driving newsvendor behavior. Such information should help identify methods to foster optimal behavior. We also hope that our research will help encourage other scholars to consider ways in which established newsvendor results may be applicable to other settings.

## Supporting information

S1 File Subject instructions and comprehension questions. (DOCX)

S1 Raw data Raw data from laboratory experiment. (XLSX)

S1 Text Individual subject level behavior. (DOCX)

S1 Fig Sequence of choices by subject in low cost newsvending treatment. (TIF)

S2 Fig Sequence of choices by subject in high cost newsvending treatment. (TIF)

S3 Fig Sequence of choices by subject in high inventory price gouging treatment. (TIF)

S4 Fig Sequence of choices by subject in low inventory price gouging treatment. (TIF)
