# Supplementary material for: Pull-to-center is not just for newsvendors
Source: PLoS One. 2022 Feb 22;17(2):e0264183. doi: 10.1371/journal.pone.0264183 (PMC8863238; doi:10.1371/journal.pone.0264183)
Supplement: S1 File — (DOCX) [file pone.0264183.s001.docx]

**Supplementary information S1**

**Subject directions and comprehension questions**

The instructions and the comprehension questions for the low cost newsvendor treatment and high inventory price gouging are presented. Test inside {} was not observed by the subjects. The other versions are similar except for minor changes and are available from the authors upon request.

Instructions {for low cost newsvendor}

Your task is to make a number of decisions under uncertainty. You have to decide how much inventory to order.

You will play 100 rounds with identical activities:

1. At the beginning of each round you receive a fixed endowment of 300 ECU.

2. You place an order, which should be from 0 to 100.

3. Once you place your order, the computer randomly generates a customer demand from a range of 0 to 100, with each number in that range equally likely. The customer demand drawn for any one round is independent of the customer demand from earlier rounds. So a small or large customer demand in earlier rounds has no influence on whether the customer demand is small or large in later rounds.

4. The profit for the round is computed. There are two different cases:

• The customer demand is less than or equal to your order quantity:

Profit = Customer demand × 100 ECUs − Order quantity × 25 ECUs + 300 ECUs

• The customer demand is greater than your order quantity:

Profit = Order quantity × 100 ECUs − Order quantity × 25 ECUs + 300 ECUs

Thus, your profit in each round is the minimum of your order quantity and the customer demand times the selling price of 100 ECU, minus your order quantity times the purchase cost of 25 ECU, plus 300 ECU.

At the end of the experiment you will be paid your cumulative earnings at the rate of $1 = 12,500 ECU.

Comprehension questions

Q1. Suppose you ordered 50 units and the computer generated a customer demand of 10 units.

What is your profit (in ECUs) in this round?

- -750
- 50 (correct)

Q2. Suppose you ordered 50 units and the computer generated a customer demand of 90 units.

What is your profit (in ECUs) in this round?

- 3250
- 4050 (correct)

Q3. How many rounds will you complete?

- 1
- 25
- 100 (correct)

Instructions {for high inventory price gouging}

Your task is to make a number of decisions under uncertainty. You have to decide what price to charge.

You will play 100 rounds with identical activities:

1. At the beginning of each round you receive a fixed endowment of 2,000 ECU.

2. You set a price, which should be from 0 to 100.

3. Once you set your order, the computer randomly generates a maximum allowed price from a range of 0 to 100, with each number in that range equally likely. The maximum allowed price drawn for any one round is independent of the maximum allowed price from earlier rounds. So a small or large maximum allowed price in earlier rounds has no influence on whether the maximum allowed price is small or large in later rounds.

4. The profit for the round is computed. There are two different cases:

• The maximum allowed price is greater than or equal to your price:

Profit = 75 × Price in ECUs + 300 ECUs

• The maximum allowed price is less than your price:

Profit = 75 × Price in ECUs – 100 × (Price in ECUs – Maximum allowed price) + 300 ECUs

Thus, your profit in each round is your price in ECU times 75 units of inventory, minus 100 ECUs for each ECU you charge above the maximum allowed price, plus 300 ECU.

At the end of the experiment you will be paid your cumulative earnings at the rate of $1 = 12,500 ECU.

Comprehension questions

Q1. Suppose you set a price of 50 and the computer generated a maximum allowed price of 10.

What is your profit (in ECUs) in this round?

- -750
- 50 (correct)

Q2. Suppose you set a price of 50 and the computer generated a maximum allowed price of 90.

What is your profit (in ECUs) in this round?

- 3250
- 4050 (correct)

Q3. How many rounds will you complete?

- 1
- 25
- 100 (correct)
