# Supplementary material for: Pull-to-center is not just for newsvendors
Source: PLoS One. 2022 Feb 22;17(2):e0264183. doi: 10.1371/journal.pone.0264183 (PMC8863238; doi:10.1371/journal.pone.0264183)
Supplement: S1 Text — (DOCX) [file pone.0264183.s003.docx]

**Supplementary information S3**

**Individual subject level plot of behavior**

Figs S3.1 – S3.4 provide the sequence of choices of each individual subject by treatment. The mean choice is reported for each subject along with the p-value associated with testing if the subject’s average choice differed from the optimal level. The correlation between the subject’s choice in one period and the ex-post optimal choice in the previous period is also shown. Demand chasers in the newsvending treatments and price chasers in the price gouging treatment are those for whom this correlation exceeds 0.195 as this is the cut-off for statistical significance at the 0.05 level when a subject makes 100 choices. Demand and price chasers are denoted with a C.
