# Supplementary figures and images for: Pull-to-center is not just for newsvendors
Source: PLoS One. 2022 Feb 22;17(2):e0264183. doi: 10.1371/journal.pone.0264183 (PMC8863238; doi:10.1371/journal.pone.0264183)

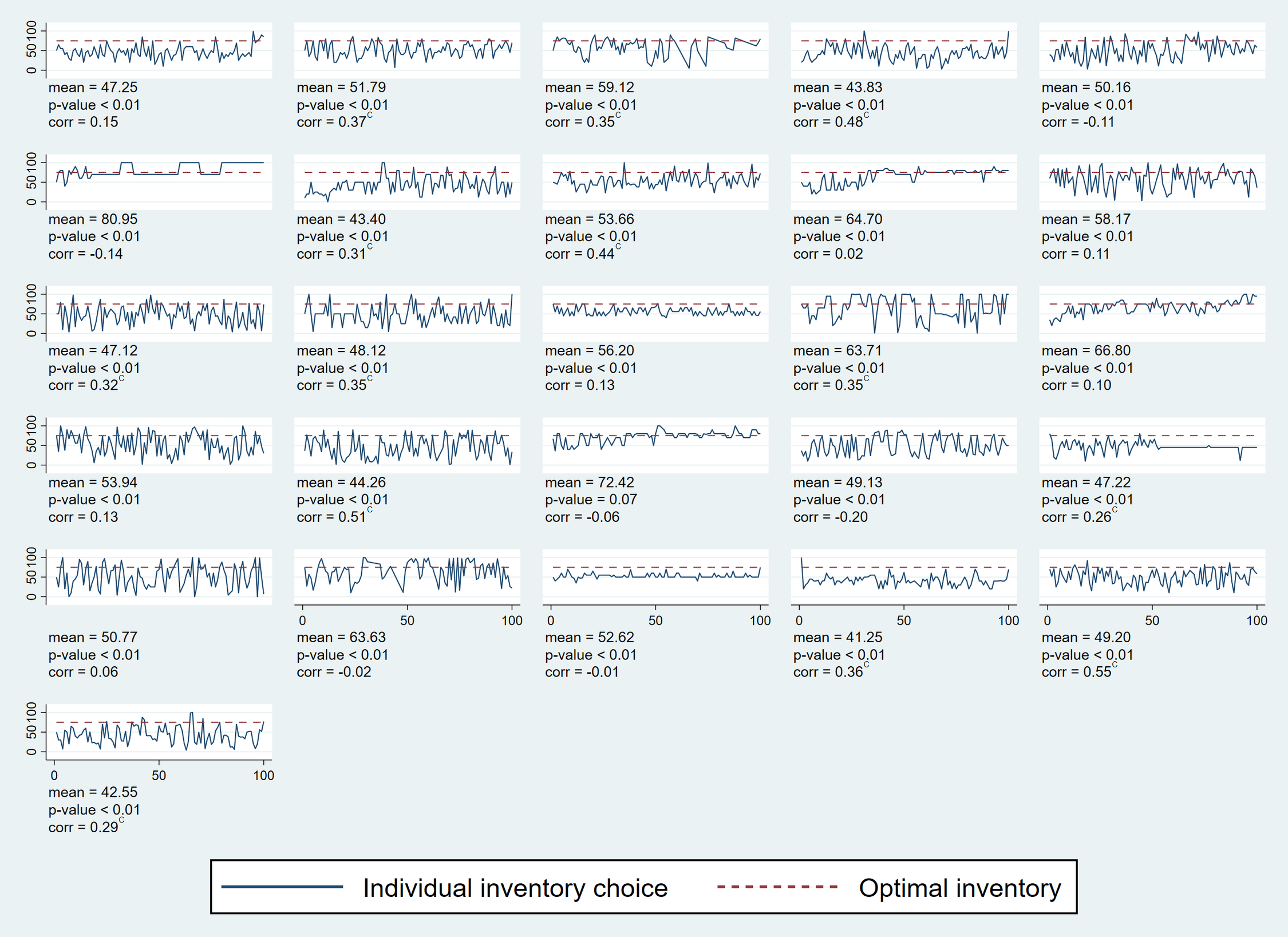

Supplement: S1 Fig — (TIF) [file pone.0264183.s004.tif]

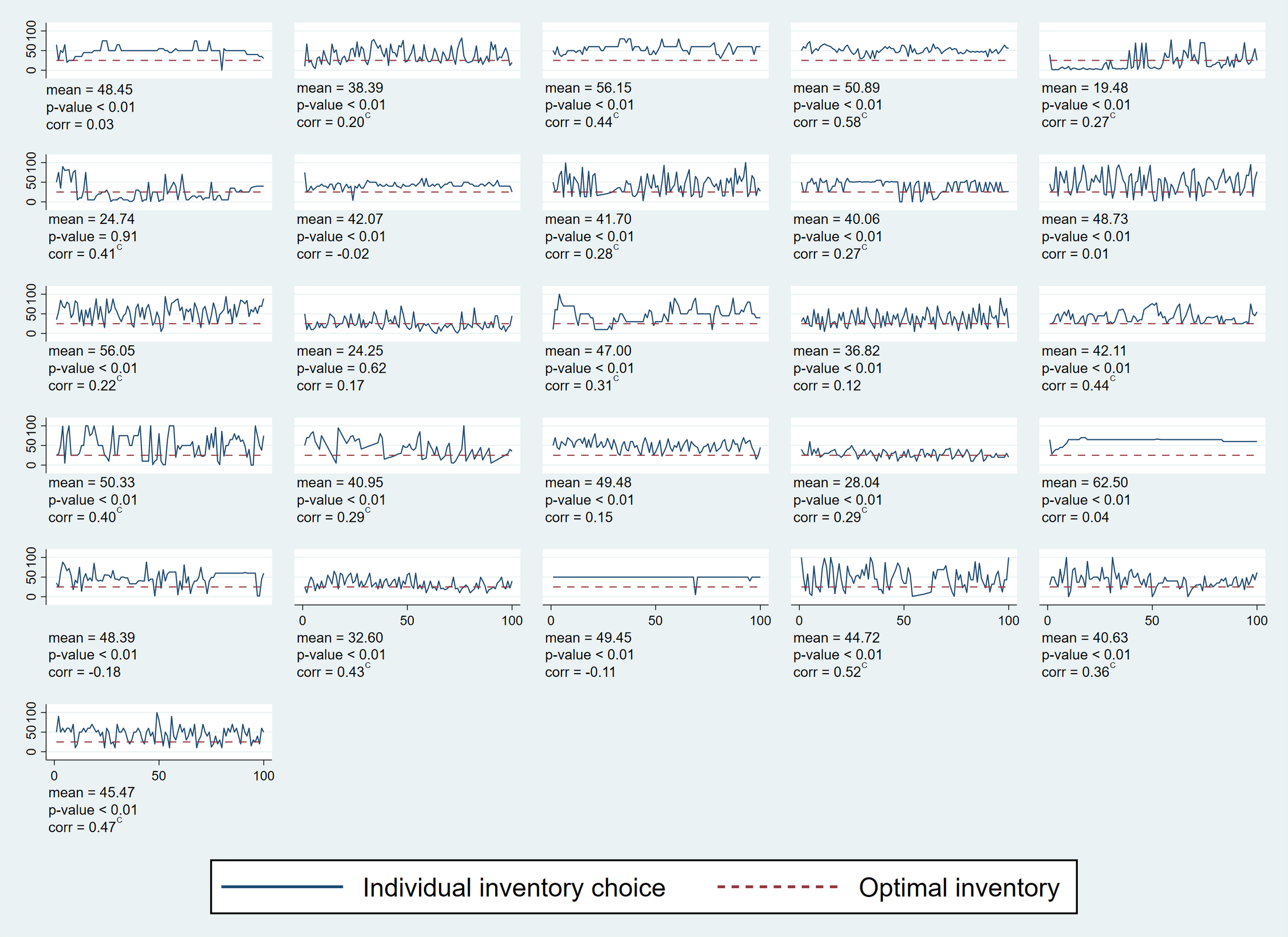

Supplement: S2 Fig — (TIF) [file pone.0264183.s005.tif]

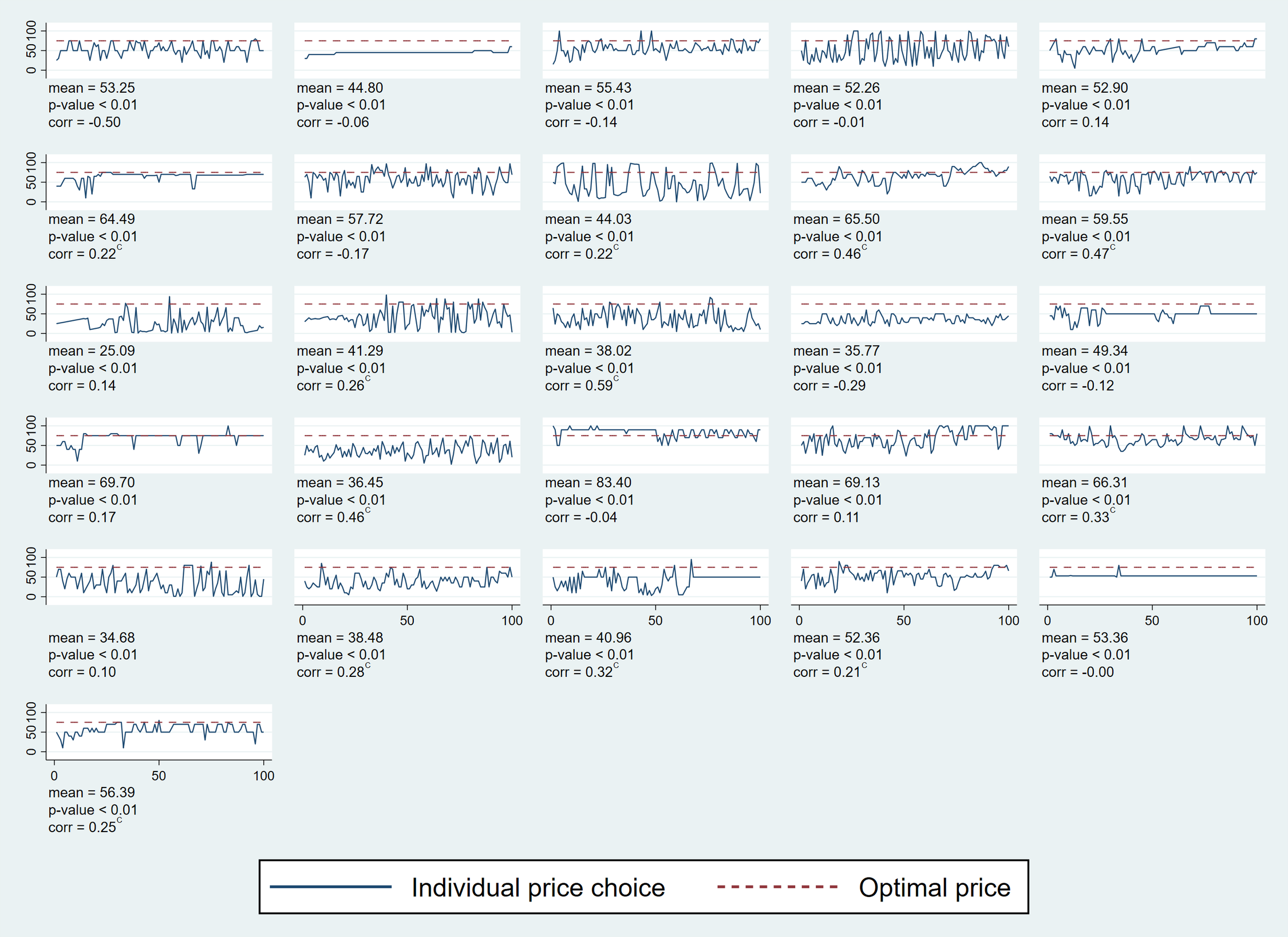

Supplement: S3 Fig — (TIF) [file pone.0264183.s006.tif]

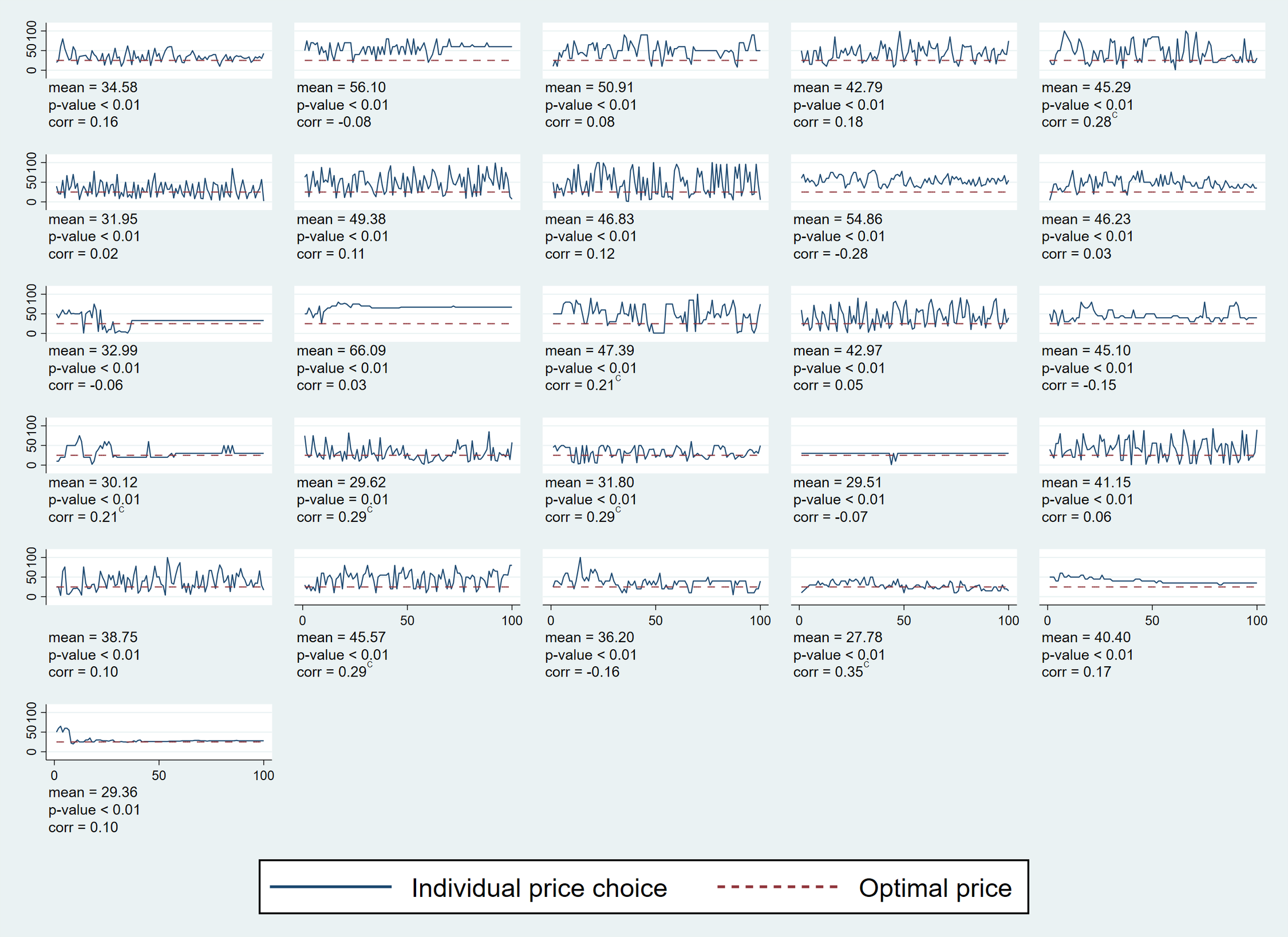

Supplement: S4 Fig — (TIF) [file pone.0264183.s007.tif]
